# Genome-wide linkage scan for loci influencing plasma triglyceride levels

**DOI:** 10.1186/s12919-018-0137-6

**Published:** 2018-09-17

**Authors:** Juan M. Peralta, Nicholas B. Blackburn, Arthur Porto, John Blangero, Jac Charlesworth

**Affiliations:** 1 South Texas Diabetes and Obesity Institute, University of Texas at the Rio Grande Valley, One West University Blvd, Brownsville, TX 78520 USA; 2 Menzies Institute for Medical Research, University of Tasmania, 17 Liverpool Street, Hobart, TAS 7000 Australia

## Abstract

We conducted a genome-wide linkage scan to detect loci that influence the levels of fasting triglycerides in plasma. Fasting triglyceride levels were available at 4 time points (visits), 2 pre- and 2 post-fenofibrate intervention. Multipoint identity-by-descent (MIBD) matrices were derived from genotypes using IBDLD. Variance-component linkage analyses were then conducted using SOLAR (Sequential Oligogenic Linkage Analysis Routines). We found evidence of linkage (logarithm of odds [LOD] ≥3) at 5 chromosomal regions with triglyceride levels in plasma. The highest LOD scores were observed for linkage to the estimated genetic value (additive genetic component) of the log-normalized triglyceride levels in plasma. Our results suggest that a chromosome 10 locus at 37 cM (LOD_pre_ = 3.01, LOD_post_ = 3.72) influences fasting triglyceride levels in plasma regardless of the fenofibrate intervention, and that loci in chromosomes 1 at 170 cM and 4 at 24 cM ceases to affect the triglyceride levels when fenofibrate is present, while the regions in chromosomes 6 at 136 to 162 cM and 11 at 39 to 40 cM appear to influence triglyceride levels in response to fenofibrate.

## Background

Triglyceride levels in plasma are highly heritable traits that have been consistently associated with obesity, cardiovascular disease, coronary heart disease, Type 2 diabetes, and metabolic disease. The genetics of triglycerides has been the subject of extensive research, including both genome-wide association and genome-wide linkage studies in a variety of cohorts.

As a result, several loci have been either associated or linked with changes in triglyceride levels. Li et al. [1] found linkage between serum triglyceride levels and chromosome 7 at 174 cM (7:174 cM, logarithm of odds [LOD] = 3.52) and suggestive linkage to 20:101 cM (LOD = 2.34), 13:111 cM (LOD = 2.00), and 9:104 cM (LOD = 1.90) in a sample of 1514 subjects from 320 obese white nuclear families. In a study of atherogenic dyslipidemia, Yu et al. [2] reported linkage of triglyceride levels to 11:99 cM (LOD = 3.34) and 17:57 cM (LOD = 3.44) in a Turkish family cohort, and to 8:54 cM (LOD = 3.08) in an extended mixed family cohort with European, Finnish, and Turkish/Mediterranean ancestry. A study of Australian dizygotic twins by Middelberg et al. [3] found linkage between triglyceride levels and 7:65 cM (LOD = 2.94), 4:62 cM (LOD = 2.26), and X:81 cM (LOD = 2.01), whereas a study of Mexican American families from Coletta et al. [4] found linkage of triglyceride levels to 12:24 cM (LOD = 3.8).

Besides design and methodological differences, those linkage studies share the characteristic of being observational studies. The Genetics of Lipid Lowering Drugs and Diet Network (GOLDN) study [5] conducted an intervention in a cohort of families of European ancestry with fenofibrate, a drug known to decrease triglyceride levels in plasma. GOLDN data were collected at 4 time points (2 before the fenofibrate intervention and 2 after it) providing an opportunity to investigate the genetic effects associated with the response to fenofibrate administration. Hidalgo et al. [6] found, using GOLDN data, a strong linkage signal for changes in low-density lipoprotein cholesterol (LDL-C) near 7:108 cM (LOD = 5.17) and for changes in high-density lipoprotein cholesterol (HDL-C) near 10:37 cM (LOD = 4.75), but reported no evidence of linkage to either total cholesterol or triglyceride levels.

In this study, we conduct genome-wide linkage scans to map loci that influence fasting triglyceride levels in plasma before and after fenofibrate was administered to GOLDN participants. To do so, we follow a different approach than the one used by Hidalgo et al. [6]. We used IBDLD [7] to estimate multipoint identity by descent and empirical kinship information from genome-wide single-nucleotide polymorphism (SNP) association data, followed by linkage analysis using the variance-component framework built into SOLAR (Sequential Oligogenic Linkage Analysis Routines) [8].

## Methods

### Data set

We used real data from the GOLDN study that was made available to participants of the GAW20. Specifically, SNP dosages from 822 individuals genotyped at 718,407 loci, phenotypes from 1106 individuals, and genealogies for 4151 individuals in 188 families. Quality control of the genotype and genealogical information was conducted using PREST-plus [9], to assess the agreement of genotype-derived pairwise relatedness inferences with the corresponding genealogy.

### Trait and covariates

Fasting triglyceride (TG) levels in plasma (mg/dL) from peripheral blood, drawn at visits 1 and 2 (pre-) and at visits 3 and 4 (post-fenofibrate intervention), were used to derive phenotypes consisting of the averaged and log-normalized pre (log_tg_pre) and post (log_tg_post) fenofibrate TG levels. In addition, we used the additive genetic component of the phenotypic variance (estimated genetic value [EGV]) of log-normalized pre (egv_log_tg_pre) and post (EGV_log_tg_post) TG levels, as described by Porto et al. [10]. Age, sex, and their interactions, together with smoking status and recruitment center, were included as covariates in all of our linkage models. To account for the possibility of local substructure or stratification we also included the first 4 principal components, estimated from scored genotypes in founders and projected to all individuals, as covariates into our linkage models.

### Simulated phenotypes

One thousand heritable traits (SIMQTs) were simulated using SOLAR (version 8.1.1) [8] with zero mean, unit variance, and a 35% heritability, but not linked to any real loci.

### Physical and genetic maps

Physical coordinates and annotations for genes and marker loci were set to be relative to release 19 of the human genome (hg19) from UCSC. Genetic coordinates were interpolated, accounting for the local base pair/cM rate, from a sex-average combined physical and genetic map [11].

### Multipoint identity-by-descent and empirical kinship estimation

Multipoint estimates of identity-by-descent (MIBD) for each SNP locus and chromosome-wide empirical kinship estimates were obtained using IBDLD (version 3.33) [7] using the genotypes at marker loci remaining after a stringent linkage disequilibrium (LD)-based pruning of r^2^ ≥ 0.9 followed by a filter of minor allele count (MAC) > 5 using PLINK (version 1.90p) [12]. A genome-wide empirical kinship matrix was then constructed as the whitened transform of the weighted average of the autosomal chromosome-wide empirical kinship matrices, using hg19 chromosome lengths (in base pairs) as weights. MIBD matrices to be used for the linkage scan were obtained from IBDLD [7] estimates at the marker loci situated closest to the cM integer units in the genetic map of each chromosome, using all available SNPs within a 10-cM window around it.

### Variance component linkage analysis

The pedigree-based multipoint variance component approach built into SOLAR [8] was used to evaluate the linkage between loci in a genetic map at the cM resolution and the log-normalized pre- and post-fenofibrate TG levels, as well as their respective estimated genetic values.

## Results

Two individuals were excluded from downstream analyses based on genealogy mismatches (see [13]). One individual, 8078 (family 333), a known monozygotic twin of 2921, was also excluded to guard against the artificial inflation of heritability estimates. Of the initial 718,407 SNPs, 313,728 did not pass our LD filter and 29,047 did not pass our MAC filter. The remaining 375,632 SNPs were used in the estimation of MIBD at 3561 loci in our genetic map.

Heritability estimates for the TG level in plasma before and after the fenofibrate intervention, and across SIMQTs ranged between 33 and 48% (Table 1). Blackburn et al. [13] found little differences (< 5%) in the heritability estimates when using an IBDLD-derived empirical kinship instead of pedigree-derived kinship coefficients (which yielded lower heritability estimates). To be conservative, we used the pedigree-based kinship estimates in our linkage models.

**Table 1:** Heritability estimates

Trait	Heritability	Standard error	Significance
log_tg_pre	0.48	0.07	2.1 × 10^− 14^
log_trr_post	0.41	0.08	7.2 × 10^−9^
egv_log_tg_pre	1.00	–	–
egv_log_trr_post	1.00	–	–
SIMQT^a^	0.33	0.06	3.4 × 10^−4^

The heritability of estimated genetic values (egv_log_tg_pre and egv_log_tgr_post) is, by definition, equal to 1. The sample size of log_tg_pre (*n* = 817) was 5% higher than for log_tg_post (*n* = 774)

^a^average from 1000 simulations

**Fig. 1:**
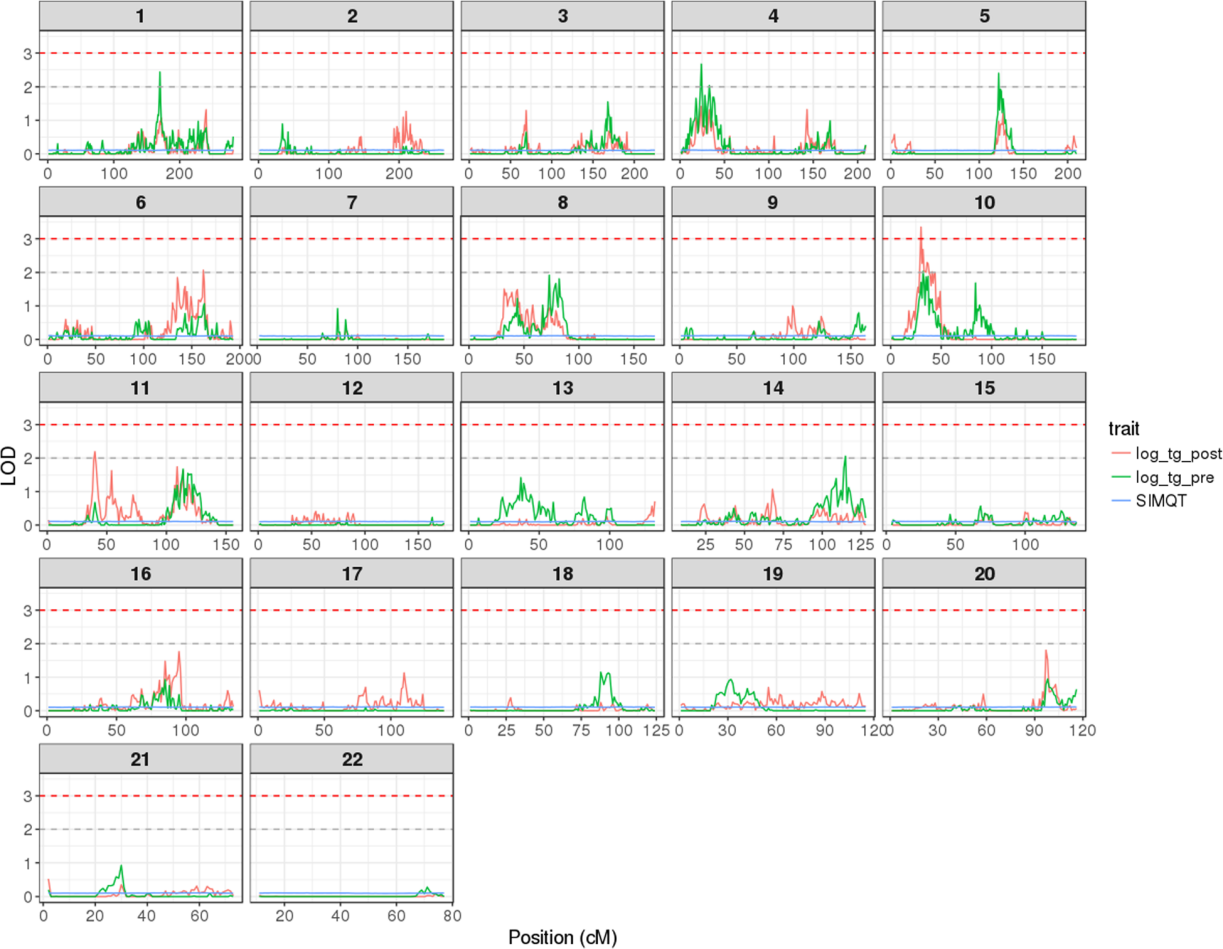
Genome-wide linkage scan plot of triglyceride levels in plasma. Linkage was defined as LOD ≥3 (horizontal red line); suggestive linkage was defined as 2 ≤ LOD < 3 (horizontal gray line); SIMQT is the average LOD score across 1000 simulations

**Fig. 2:**
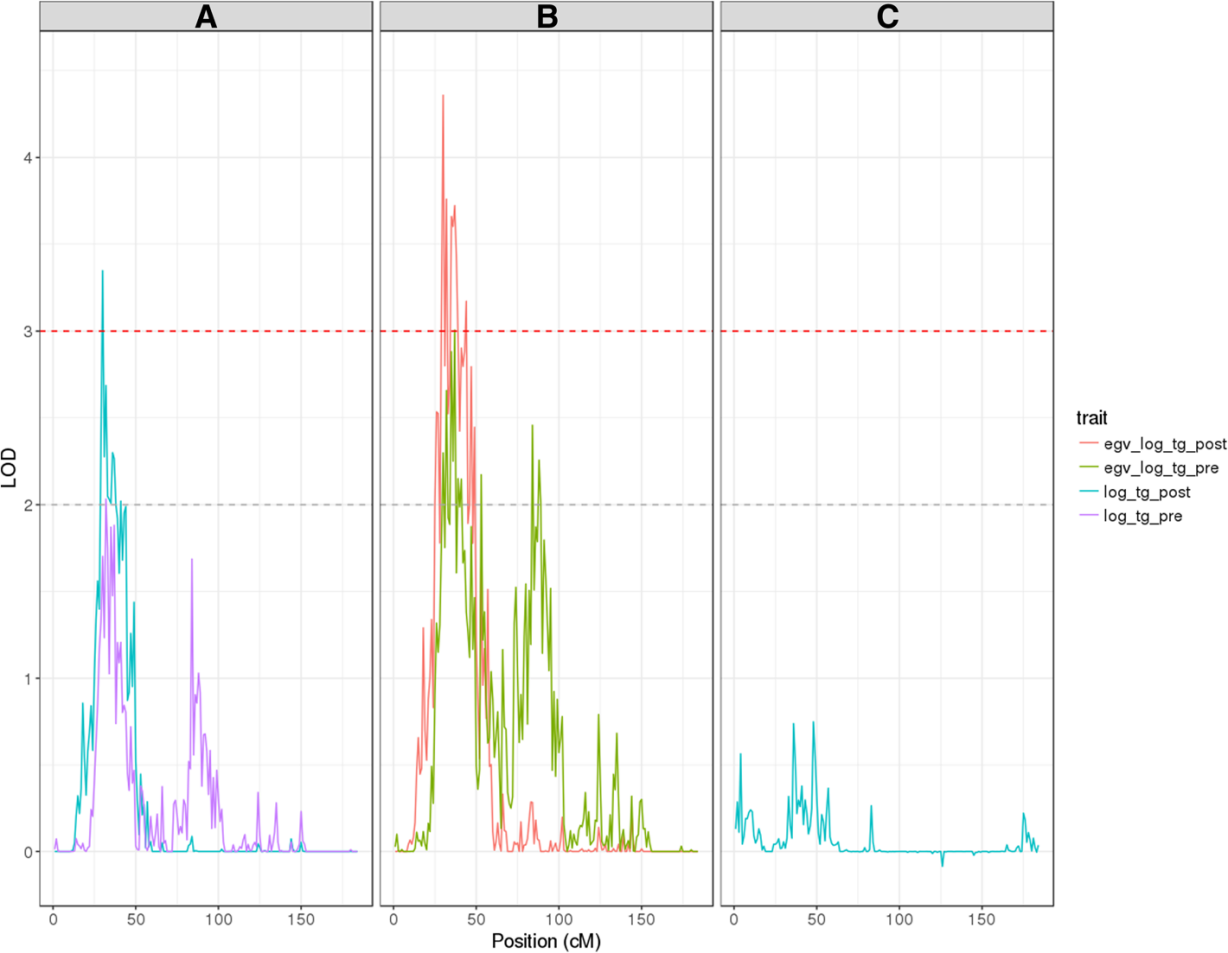
Linkage scans from chromosome 10 under 3 different models. Expanded view of the linkage scan of chromosome 10 for (**a**) triglyceride levels pre- and post-fenofibrate treatment (log_tg_pre and log_tg_post); **b** their respective estimated genetic values; and **c** the post-fenofibrate TG levels after removal of its additive genetic component, as estimated by its estimated genetic value

Linkage was detected for log_tg_post in chromosome 10 at 30 cM, with a peak LOD score of 3.35, within a 12-cM region of LOD ≥2 support (Fig. 1; Table 2). In addition, 2 suggestive linkage signals (LOD ≥2) were observed at 6:162 cM and 11:40 cM (Table 2). The linkage analysis of egv_log_tg_post yielded stronger LOD score peaks of 4.36 at 10:30 cM, 3.24 at 6:162 cM, and 3.34 at 11:40 cM (Fig. 2; Table 3). Only signals suggestive of linkage with log_tg_pre were observed (Table 2). One of these mapped to the locus at 10:32 cM, close to the region linked to log_tg_post. Significant evidence of linkage was observed for log_tg_pre at 1:170 cM, 4:24 cM, and 5:122 cM (Fig. 1: Table 2). The analysis of egv_log_tg_pre improved the magnitude of the LOD score peaks observed for log_tg_pre, at 1:170 cM and 4:24 cM, beyond the LOD ≥3 threshold for linkage (Fig. 2; Table 3). Our results also included a number of suggestive egv_log_tg_pre and egv_log_tg_post loci (data not shown).

**Table 2:** Loci linked or exhibiting suggestive linkage to fasting triglyceride levels in plasma

Trait	Chromosome	cM	LOD	h2r	h2q1
log_tg_pre	1	170	2.43	0.20	0.40
	4	24	2.67	0.12	0.44
	4	33	2.04	0.15	0.41
	5	122	2.40	0.15	0.42
	10	32	2.03	0.18	0.41
	14	115	2.06	0.15	0.42
log_tg_post	6	162	2.07	0.09	0.42
	10	29	2.36	0.11	0.43
	**10**	**30**	**3.35**	**0.05**	**0.51**
	10	31	2.27	0.10	0.43
	10	32	2.69	0.08	0.48
	10	33	2.05	0.12	0.43
	10	34	2.03	0.13	0.40
	10	35	2.01	0.13	0.39
	10	36	2.30	0.11	0.43
	10	37	2.26	0.10	0.45
	10	38	2.00	0.12	0.41
	10	41	2.02	0.11	0.43
	11	40	2.19	0.07	0.48

Linkage was defined as LOD ≥3 (in bold); suggestive linkage was defined as 2 ≤ LOD < 3. *h2r*, Residual trait heritability; *h2q1*, heritability of the quantitative trait locus

**Table 3:** Loci linked to the estimated genetic values of triglyceride levels in plasma

Trait	Chromosome	cM	LOD	h2r	h2q1
egv_log_tg_pre	1	170	3.05	0.63	0.37
	4	24	3.08	0.57	0.43
	10	37	3.01	0.54	0.46
egv_log_tg_post	6	136	3.02	0.57	0.43
	6	157	3.21	0.57	0.43
	6	162	3.24	0.60	0.40
	10	29	3.15	0.60	0.40
	10	30	4.36	0.53	0.47
	10	32	3.76	0.52	0.48
	10	35	3.66	0.53	0.47
	10	36	3.60	0.52	0.48
	10	37	3.72	0.50	0.50
	10	38	3.43	0.54	0.46
	10	44	3.17	0.65	0.35
	11	39	3.29	0.54	0.46
	11	40	3.34	0.49	0.51

Linkage was defined as LOD ≥3. *h2r*, Residual trait heritability; *h2q1*, heritability of the quantitative trait locus

To determine whether our linkage analyses were biased by the capture of short-range identity-by-descent (IBD) instead of the expected long-range MIBD estimates, we shifted the point of MIBD estimation by half a centimorgan in the genetic map. Then we estimated new MIBD matrices and repeated the linkage analyses of the log_tg_pre and log_tg_post traits. The resulting linkage scans contained the same features as those appearing in Fig. 1, with moderate variation in the magnitude of the LOD score peaks (data not shown). The most relevant difference was that with the half centimorgan shift the suggestive peaks for log_tg_pre at 10:30 cM and at 5:122 cM reached the LOD = 3 threshold.

All evidence of linkage disappeared with the removal of the genetic signal from log_tg_post, accomplished by the introduction of egv_log_tg_post as covariate into its linkage model (see Fig. 2).

Across a thousand SIMQT linkage scans we observed 1 scan with LOD scores ≥3.35, 3 scans with LOD scores ≥3, and 33 scans with LOD scores ≥2. The maximum LOD score observed in the averaged linkage scan from all SIMQTs was 0.119 (mean = 0.106, SD = 0.004).

## Discussion

Hidalgo et al. [6] reported no linkage in their analysis of TG levels, using a different approach than the one we followed here. They relied primarily on MERLIN to both estimate IBD matrices and conduct linkage analyses. This methodological choice has 2 limitations not present in our study. First, the use of MERLIN requires large and informative pedigrees to be reduced in complexity, resulting in a loss of both information and power. Second, and possibly more important, it limited the number of loci that Hidalgo et al. were able to use for IBD inference. Only 10 sets of approximately 3000 SNPs each were used to perform “linkage analyses in independent overlapping intervals of the genome” [6] from 729,490 SNPs that were available to them for analysis. By using IBDLD [7] we were able to leverage the information contained in more than 375,632 SNPs to derive our MIBD estimates at the 3561 loci that were used in our linkage analyses. IBD was estimated using all available marker data within 10-cM windows centered around the locus selected for any given MIBD matrix, an approach that we believe yielded more robust and informative IBD estimates. When we shifted our selection of markers by half a centimorgan, our linkage inferences did not change.

We found linkage between the fasting TG levels in plasma after the intervention with fenofibrate (log_tg_post) and a locus that maps to chromosome 10 at 30 cM (LOD = 3.35; see Table 2). We also discovered evidence suggestive of linkage at 18 other loci (see Table 2). To assess the relevance of these loci in the response to the fenofibrate challenge, while increasing our power to detect linkage (see Porto et al. [10]), we decided to focus on the purely additive genetic component of the TG levels, as estimated by their estimated genetic values. By introducing the estimated genetic values as covariates into our linkage models we effectively removed the additive genetic component from the phenotype. The results we obtained using these models were devoid of any sign of linkage (see Fig. 2); an indication that the features from Fig. 1 were not a random artifact and were driven by the heritable component of the analyzed trait. This encouraged us to search the genome directly for linkage with the TG level estimated genetic values. As a result for most of the loci the LOD scores reached the linkage threshold, as shown in Table 3. Based on the linkage analysis of the estimated genetic values it appears that loci at 1:170 cM and 4:24 cM cease to have an influence on the TG levels in the presence of fenofibrate, whereas the regions at 6:136 cM to 6:162 cM and 11:39 cM to 11:40 cM appear to influence TG levels in response to fenofibrate. The region at 10:29 cM to 10:44 cM appears to affect TG levels regardless of the fenofibrate intervention. However, it would be premature to interpret these results as evidence of a possibly differentiated subject response to the fenofibrate treatment. A rigorous exploration of the response to the drug treatment, while an interesting research avenue, was beyond the scope of this study.

There are precedents in the literature for some of our findings. The analysis from Liu et al. [14] found suggestive linkage signals for combined TG/HDL levels within the 6:129 cM to 6:155 cM region in the Framingham Heart Study cohort. Middelberg et al. [3] reported linkage to 4:62 cM, in the vicinity of the feature that contains our 4:24 cM linkage (see Fig. 1). While the linkage to 11:99 cM described by Yu et al. [2] is far from our 11:39 cM to 11:40 cM signal, we did find suggestive linkage to the estimated genetic value of post-fenofibrate treatment at 11:114 cM (LOD = 2.94). Finally, Hidalgo et al. [6] described linkage to 10:30 cM to 10:50 cM for changes in HDL-C.

## Conclusions

We demonstrated that conducting linkage scans with MIBD matrices estimated from dense SNP loci is feasible. Focusing our analyses on the additive genetic component of the trait allowed us to improve our power to detect linkage. Our results identified loci that appear to influence TG levels in plasma and seem consistent with a differential response to the presence of fenofibrate.
